# Urogenital Cancers: New Molecular and Translational Aspects on Carcinogenesis and Treatments

**DOI:** 10.3390/biomedicines13112678

**Published:** 2025-10-31

**Authors:** Anna Perri, Vittoria Rago, Silvia Di Agostino

**Affiliations:** 1 Department of Pharmacy, Health and Nutritional Sciences, University of Calabria, 87036 Rende, Italy; anna.perri@unical.it (A.P.); vittoria.rago@unical.it (V.R.); 2 Department of Health Sciences, Magna Graecia University of Catanzaro, 88100 Catanzaro, Italy

Over the past twenty years, owing to the spread of screening programs, the incidence of urogenital cancers has shown an increasing trend, especially for certain types of cancer, such as endometrial and ovarian cancers in women and prostate cancer in men [1]. Urogenital cancers include multiple malignancies of the urogenital tract, and each type of tumor exhibits different gender-related risk factors and unique mechanisms of progression and metastasis, therefore requiring different and specific therapeutic approaches [2,3,4,5,6].

An early and as accurate as possible diagnosis, also throughout the stratification by molecular biomarkers, allows for therapeutic intervention that is as targeted as possible to the pathological and molecular characteristics of the tumor [7,8,9,10,11,12,13].

Molecular markers play an important role in early tumor diagnosis, patient stratification, development of new therapies, disease monitoring, and prognosis [14,15,16,17]. However, reliable biomarkers for some urogenital tumors are still lacking, making therapies less effective [18,19,20,21,22].

Briefly, the primary objective of the “Urogenital Cancers: New Molecular and Translational Aspects on Carcinogenesis and Treatments” Special Issue was to discuss the most current aspects of these heterogeneous tumors, exploring novel molecular mechanisms that could shed light on the identification of specific targets that may be useful for precision therapies. The Special Issue also focused on contributions describing novel treatment and diagnosis approaches for urogenital tumors.

The contributions included three reviews and eight original research articles, which revealed that the most studied malignancies were bladder, prostate, and ovarian cancers. The most frequently discussed topics included the metastatic mechanisms of these tumors, the identification of specific biomarkers for tumor progression and response to therapy, and the validation of new therapeutic strategies to bypassing resistance to conventional therapies [23,24,25,26].

Most of the manuscripts published in this Special Issue fall into the following three broad categories:

Biomarkers, which, as previously mentioned, are the starting point for accurate diagnosis, monitoring the effectiveness of therapy, and evaluating patient outcomes. The studies provided important findings, for example developing a biomarker-based nomogram to predict response to neoadjuvant chemotherapy of patients with muscle-invasive bladder cancer and their relapse risk [27]. Transcriptomic analysis from bone-metastatic prostate cancer [28] or DNA methylation patterns in testicular germ cell tumors [29] have shown promising key molecular targets for the development of novel therapeutic approaches.

Therapy is the biggest goal in cancer research, and some studies have reported new approaches, for example in bladder cancer and prostate cancer [30,31,32].

Nutrients, including selenium (Se) and zinc (Zn) could impact the survival of patients, and the role of Se and Zn in cancer outcome is still sparsely investigated. Selenium and zinc were investigated in the blood and serum of kidney cancer patients, showing that low selenium concentrations and combined selenium–zinc deficiencies were associated with significantly worse 10-year survival [33]. These findings suggest that micronutrient profiling may inform new preventive or supportive interventions.

The bulk of papers published in this Special Issue highlighted the importance of genomic instability, DNA damage and repair mechanisms, and drug resistance for improving diagnostic methods; understanding the mechanisms of progression and metastasis of urogenital tumors; and, consequently, developing appropriate treatments for patients.

In the future, we hope that factors such as dietary habits, the influence of the microbiome, and viral infections will provide further insights into this type of tumor. Furthermore, more intensive study of the intratumoral heterogeneity of each tumor and its influence on treatment success would be desirable.

Overall, the diverse scientific contributions highlighted that integrating multidisciplinary approaches will be essential for addressing the therapeutic challenges posed by these heterogeneous and complex neoplasms and improving patient care in the era of personalized molecular medicine.
